# Using a Safety Planning Mobile App to Address Suicidality in Young People Attending Community Mental Health Services in Ireland: Protocol for a Pilot Randomized Controlled Trial

**DOI:** 10.2196/44205

**Published:** 2023-02-21

**Authors:** Ruth Melia, Kady Francis, Jim Duggan, John Bogue, Mary O'Sullivan, Karen Young, Derek Chambers, Shane J McInerney, Edmond O'Dea, Rebecca Bernert

**Affiliations:** 1 Department of Psychology University of Limerick Limerick Ireland; 2 School of Psychology, University of Galway Galway Ireland; 3 Health Service Executive Community Healthcare Mid West Limerick Ireland; 4 Health Service Executive Community Healthcare West Galway Ireland; 5 Insight Centre for Data Analytics University of Galway Galway Ireland; 6 College of Engineering and Informatics, University of Galway Galway Ireland; 7 Health Service Executive Dublin Ireland; 8 Department of Psychiatry University of Galway Galway Ireland; 9 Department of Psychiatry and Behavioral Science Stanford University Palo Alto, CA United States

**Keywords:** suicide prevention, mobile health, mHealth, safety planning, SafePlan, mobile phone

## Abstract

**Background:**

Over 700,000 people die by suicide annually, making it the fourth leading cause of death among those aged 15-29 years globally. Safety planning is recommended best practice when individuals at risk of suicide present to health services. A safety plan, developed in collaboration with a health care practitioner, details the steps to be taken in an emotional crisis. SafePlan, a safety planning mobile app, was designed to support young people experiencing suicidal thoughts and behaviors and to record their plan in a way that is accessible immediately and in situ.

**Objective:**

The aim of this study is to assess the feasibility and acceptability of the SafePlan mobile app for patients experiencing suicidal thoughts and behaviors and their clinicians within Irish community mental health services, examine the feasibility of study procedures for both patients and clinicians, and determine if the SafePlan condition yields superior outcomes when compared with the control condition.

**Methods:**

A total of 80 participants aged 16-35 years accessing Irish mental health services will be randomized (1:1) to receive the SafePlan app plus treatment as usual or treatment as usual plus a paper-based safety plan. The feasibility and acceptability of the SafePlan app and study procedures will be evaluated using both qualitative and quantitative methodologies. The primary outcomes are feasibility outcomes and include the acceptability of the app to participants and clinicians, the feasibility of delivery in this setting, recruitment, retention, and app use. The feasibility and acceptability of the following measures in a full randomized controlled trial will also be assessed: the Beck Scale for Suicide Ideation, Columbia Suicide Severity Rating Scale, Coping Self-Efficacy Scale, Interpersonal Needs Questionnaire, and Client Service Receipt Inventory. A repeated measures design with outcome data collected at baseline, post intervention (8 weeks), and at 6-month follow-up will be used to compare changes in suicidal ideation for the intervention condition relative to the waitlist control condition. A cost-outcome description will also be undertaken. Thematic analyses will be used to analyze the qualitative data gathered through semistructured interviews with patients and clinicians.

**Results:**

As of January 2023, funding and ethics approval have been acquired, and clinician champions across mental health service sites have been established. Data collection is expected to commence by April 2023. The submission of completed manuscript is expected by April 2025.

**Conclusions:**

The framework for Decision-making after Pilot and feasibility Trials will inform the decision to progress to a full trial. The results will inform patients, researchers, clinicians, and health services of the feasibility and acceptability of the SafePlan app in community mental health services. The findings will have implications for further research and policy regarding the broader integration of safety planning apps.

**Trial Registration:**

OSF Registries osf.io/3y54m; https://osf.io/3y54m

**International Registered Report Identifier (IRRID):**

PRR1-10.2196/44205

## Introduction

### Background

More than 700,000 people die by suicide every year, representing the fourth leading cause of death among 15-29-year-olds globally in 2019 [1]. In Ireland, provisional figures indicate that 340 individuals died by suicide in 2020 [2]. Approximately one-third of people who die by suicide have been in contact with mental health services in the year before their death, and approximately 1 in 5 had contact with a health professional in the month before their death [3]. Suicide prevention guidelines recommend safety planning interventions alongside follow-up treatment by mental health services for those presenting to health services at risk of suicide [4]. However, the decision to implement safety planning in practice can be influenced more by clinicians’ beliefs about their efficacy than by empirical research [5]. The transient nature of suicidal ideation [6] may challenge the feasibility of paper-based plans and suggests the need for alternative modalities.

### Suicide Risk Factors

Suicidal behavior remains extremely difficult even for experienced clinicians to predict [7], leading researchers to focus their efforts on brief interventions designed for those at immediate risk [8]. A meta-analysis of 50 years of research in the area indicated that our predictive ability has not improved in that time and called for a shift from a focus on risk factors to machine learning–based risk algorithms [9]. The application of machine learning to large data sets may offer a means of better understanding the complex processes proposed by prominent theorists in the area [10,11]. Reflective of these research findings, recent clinical best practice guidelines recommend against the use of suicide risk assessment measures to predict future suicide or repeated self-harm in young people [12] and point to the importance of comprehensive psychosocial assessment and the development of a collaboratively developed and accessible safety plan.

### Safety Planning Intervention

The safety planning intervention (SPI) developed by Stanley and Brown [13] is a collaborative safety plan developed with a trained practitioner and recorded using a paper-based document. It is a brief structured intervention widely used in health systems and is often cited as the best practice in the treatment of patients with a high risk of suicide [14]. SPI is a key component across multiple treatment approaches targeting suicidal behavior, such as collaborative assessment and management of suicidality and cognitive behavioral therapy for suicide prevention. A safety plan outlines the steps to take in an emotional crisis and includes the identification of personalized warning signs, internal coping skills, people and places that provide distraction, people to contact for support, and contact details to use in a crisis. A cohort comparison trial of patients in the emergency department in US veteran hospitals presenting with suicidality found that SPI and phone follow-up significantly reduced suicidal behaviors and increased treatment engagement [13]. A recent systematic review of the effectiveness of SPI for adults experiencing suicide-related distress indicated improvements in suicidality (ideation, behaviors, and death), suicide-related outcomes (depression and hopelessness), and treatment outcomes (hospitalization) [15].

### Safety Planning Mobile Apps

Recording a safety plan on a mobile app may further enhance accessibility. The use of mobile devices by individuals at risk of suicide has been recommended by the World Health Organization [16]. The delivery of suicide prevention interventions through mobile health (mHealth) apps provides many opportunities when individuals need help the most [17]. SPI has been shown to be adaptable in its modality (digital or paper based) [15]. Further research is needed to better understand the extent to which using apps as an add-on to the treatment of patients with suicidal tendencies is feasible for clinicians and patients themselves. The MYPLAN safety planning app is undergoing clinical trial (ClinicalTrial.gov NCT02877316), and the Continuous Assessment for Suicide Prevention And Research study aims to implement a Dutch translation of the MYPLAN app alongside a self-monitoring app for suicidal patients in the Netherlands [18]. Researchers recently reported on the feasibility of using smartphone apps as treatment components for outpatients with depression and suicidal tendencies and found that mobile safety planning (using the BackUp app) was acceptable and usable for this group; however, the integration of apps into routine treatment needs to be further explored [19]. Indeed, researchers attest that effective interventions for suicidality do exist, but many patients do not receive them because implementation efforts tend to be time limited and unsystematic [20].

### Integrating mHealth in Mental Health Services

This protocol is underpinned by the research team’s previous research on the experiences of mental health professionals involved in integrating mHealth technology into existing mental health services [21]. The potential benefits of using mobile apps identified by mental health professionals include increased accessibility, increased engagement with young people, and the convenience and feasibility of a client using a mobile app during a crisis [21]. Many of the benefits identified in previous research are consistent with those asserted by mental health professionals in previous research [22]. A concern identified by professionals included the potential for the technology to be used instead of face-to-face services. Such findings are in line with previous research [23], suggesting that although professionals acknowledge their possible benefits and cost-effectiveness, health care providers are conservative about integrating mHealth technologies into everyday practice.

Mental health professionals identified the following barriers to adoption: lack of trust in the technology (privacy and confidentiality) and in the health care organization, training needs, policy guidance requirements, technical support requirements, and the need for further research. This mirrors the systematic review findings of the app literature, calling for further research before such technology is recommended or prescribed [24]. The benefits identified included the needs addressed by this technology to increase accessibility, enhance engagement, enhance the efficacy and generalization of skills acquired outside of the clinic setting, and the potential for individualized data collection to enhance treatment [24]. Similar enablers have been cited by mental health professionals in previous research [23].

### Engaging Young People

The use of mobile apps to record and store one’s safety plan may be particularly useful in supporting young people experiencing suicidal thoughts and behaviors, often simultaneously experiencing a transition to adulthood and to adult-focused services. The onset of many psychopathologies has been shown to coincide with the transition age [25,26]. Approximately 75% of all psychiatric disorders in adults start before the age of 24 years, and 50% of all psychiatric disorders start before the age of 14 years. However, there is discord in the pattern of increased risk of psychopathology in young people and mental health service use. Clinicians have previously identified the potential benefits of mobile app use in promoting engagement among young people with mental health services [21]. Approximately 64% of adolescents reported using apps to manage their mental health symptoms [27]. Indeed, 59% of health and fitness app users are aged between 18 and 34 years, indicating a higher level of engagement in health apps within this age group [28].

### Methodological Considerations

This trial is informed by a systematic review examining the effectiveness of mHealth technology tools in reducing suicide-specific outcomes, along with the review protocol [29,30]. This systematic review identified 7 studies that met the inclusion criteria. A total of 4 published articles that reported on the effectiveness of the following mobile phone apps were included: iBobbly [31], Virtual Hope Box [32], BlueIce [33], and Therapeutic Evaluative Conditioning [34]. The results demonstrated some positive impacts for individuals at an elevated risk of suicide or nonsuicidal self-injurious behaviors, including reductions in depression, psychological distress, and self-harm and increases in coping self-efficacy. However, none of the evaluated apps demonstrated the ability to significantly decrease suicidal ideation when compared with a control condition.

A significant limitation identified across all studies pertained to the heterogeneity of outcomes. Studies focused on differing suicide outcomes such as suicidal ideation as opposed to suicidal behaviors and often used differing outcome measures (ie, Beck Suicidal Intent Scale, Columbia Suicide Severity Rating Scale [C-SSRS], and Suicidal Ideation Attributes Scale). This hampered the extent to which the data could be compared and generalized. Another methodological issue is the practice of excluding individuals experiencing current suicidal thoughts or behaviors from participation. Importing empirically supported treatments for depression may not be appropriate because the trials in which efficacy was established excluded individuals who engage in suicidal behavior By embedding the trial within secondary-level mental health services, individuals at risk of suicide can participate safely while accessing mental health treatment. This methodological change is designed to reduce sample bias, enhance generalizability, and inform implementation research [20]. The evidence available indicates that suicide prevention mobile apps lead to reductions in depression, psychological distress, and self-harm in those at elevated risk of suicide or self-harm [29]. However, the pace of suicide prevention app development needs to be matched by scientifically rigorous research and the use of clinically valid methodologies that inform broader implementation of existing mental health services.

### SafePlan

SafePlan is a safety planning mobile app designed to be used as an adjunct to therapy for individuals experiencing suicidal thoughts and behaviors who access mental health services. The SafePlan app was designed and developed following significant stakeholder engagement and cocreation with young people, clinicians, suicide prevention researchers, suicide prevention policy developers, computer scientists, and human-computer interaction specialists. The SafePlan design process is described in detail elsewhere [35]. In addition to the capacity to develop and record a collaborative safety plan on the app, it also supports the completion of dialectical behavior therapy (DBT) diary cards, which was deemed appropriate. This feature was included on the advice of clinicians who noted that individuals who complete a safety plan as part of their treatment may also engage in DBT. The integration of DBT diary cards [36] can be enabled in the app’s clinical settings, in consultation with a mental health care professional, for patients who are engaged in this form of intervention. Consistent with research evaluating the Virtual Hopebox [32], the app also supports the inclusion and display of personalized Reasons for Living, such as photos, text, and videos, in visibly prominent places within the app.

This study details the first feasibility trial of the SafePlan mobile app within mental health services. Current Medical Research Council (MRC) guidance on complex interventions [37] advocates pilot trials and feasibility studies as part of a phased approach to the development, testing, and evaluation of health care interventions.

### Study Objectives

The study objectives are as follows: (1) examine the feasibility and acceptability of the SafePlan app as an adjunct to therapy for patients (aged 16-35 years) accessing secondary-level Irish mental health services and their clinicians; (2) assess the feasibility of the study procedures for both clinicians and patients within secondary-level mental health services; (3) determine the sample size required for a full randomized controlled trial (RCT) by collecting suicide outcome data at baseline, post intervention, and at 6-month follow-up; (4) determine whether the SafePlan app condition yields superior outcomes on the outcomes of suicidal ideation, suicidal behavior, and nonsuicidal self-injurious behavior at 6-month follow-up and post treatment when compared with the control condition; and (5) complete an economic analysis of the intervention.

### Research Question

Is the SafePlan app, along with the proposed outcome measures, feasible and acceptable to patients aged 16-35 years at risk of suicide and their clinicians, in secondary-level mental health services in Ireland?

The feasibility and acceptability of using the following outcome measures in a full RCT will be assessed: the Beck Scale for Suicide Ideation (BSSI), C-SSRS, Coping Self-Efficacy Scale (CSES), Interpersonal Needs Questionnaire (INQ), and Client Service Receipt Inventory (CSRI) [38-42].

Data will be collected at baseline, post intervention, and at 6-month follow-up to determine whether the SafePlan app yielded superior outcomes on primary outcomes of suicidal ideation, suicidal behavior, and nonsuicidal self-injurious behavior when compared with the control group.

- Participants: A total of 80 patients aged 16-35 years accessing secondary-level mental health services who present with current suicidal ideation, nonsuicidal self-injurious behavior, or a previous suicide attempt will be randomized to the SafePlan or control condition.
- Intervention: SafePlan is a safety planning mobile app informed by the paper-based SPI by Brown and Stanley [13]. Participants in the SafePlan condition will use the SafePlan app to record their safety plan in consultation with their mental health professional over an 8-week period as an adjunct to usual treatment.
- Comparison: The control group will receive treatment as usual (TAU) supplemented with the paper-based safety plan by Brown and Stanley [13] completed with their mental health professional.
- Outcome: The primary outcomes are feasibility outcomes and include the acceptability of the app to participants and clinicians, feasibility of delivery in this setting, recruitment, retention, and app use. Data collected on measures of suicidal ideation; suicidal behavior; and nonsuicidal self-injurious behavior at baseline, post intervention (8 weeks), and at 6-month follow-up will be used to compare changes in outcomes for the SafePlan condition relative to the control condition. A cost-outcome description will also be undertaken. Thematic analyses will be used to analyze the qualitative data gathered through semistructured interviews with patients and clinicians.

## Methods

### Design

The SafePlan study is a pilot RCT of SafePlan, a mobile-based safety planning app. A parallel group randomized design will be used with 1:1 patient allocation to the 2 treatment arms. In the control condition, participants will receive TAU supplemented with a paper-based safety plan. In the intervention condition, participants will use the SafePlan app to record their safety plan in consultation with their mental health professional, in addition to routine treatment. In both conditions, participants will receive appropriate care through their mental health team for an elevated risk of suicide throughout the study. Quantitative data will be collected at baseline, post intervention (8 weeks), and at 6-month follow-up (from baseline), with an additional safety check assessment at 4 weeks in both conditions.

### Participants

Participants will include patients aged 16-35 years who are currently accessing secondary-level mental health services (child and adolescent mental health services [CAMHS] or adult mental health Services [AMHS]). Participants who present with current suicidal ideation, nonsuicidal self-injurious behavior in the previous 6 months, or a previous self-reported suicide attempt will be recruited across 2 community health care organizations. Participants will be assessed by members of their mental health team, will be accessing treatment with an assigned key therapist for the entire duration of the intervention, and will provide appropriate consent (for participants aged 16-18 years, parental consent is also required). Key workers will inform potential participants who meet the eligibility criteria (Textbox 1) and for whom a SPI is clinically indicated. Participants’ psychiatric diagnoses will be recorded, but it will not be a cause for exclusion, as suicidality occurs across diagnostic categories.

**Participant inclusion and exclusion criteria.**

**Inclusion criteria**

- Aged 16-35 years
- Presenting with current suicidal ideation, nonsuicidal self-injurious behavior in the last 6 months, or having a lifetime history of a self-reported suicide attempt
- Currently accessing Health Service Executive community mental health services (child and adolescent mental health services or adult mental health services)
- Have been allocated a key clinician to work with the participant for the duration of the intervention
- The key clinician or treating team identify that a safety plan is clinically indicated
- They were proficient in English to allow the provision of informed consent and the completion of measures in English
- Use a smartphone and can download the SafePlan app

**Exclusion criteria**

- Indicates no suicidal ideation, nonsuicidal self-injurious behavior, or previous suicide attempts
- Are medically unfit for interview
- Are not actively engaged with mental health services
- Are unable to provide informed consent
- Are outside of the specified age range
- Level of English is not sufficient to complete outcome measures in English
- Do not have access to a smartphone

### Clinicians

Clinicians involved in providing the intervention in both arms will be mental health service professionals and members of the patient’s multidisciplinary team who have undergone specialist training in addressing suicidality in clinical practice and the implementation of safety planning and who have completed the SafePlan technical orientation and training session. Data regarding the type of treatment received will be collected across both groups from the person’s clinical file (number of face-to-face contacts, therapeutic approach used, profession of the key clinician, and prescribed medications) and the CSRI [42].

### Sample Size

There is no established gold standard for calculating the sample size for a feasibility study, as the purpose of the study determines the sample size justification. In a systematic assessment of sample sizes in pilot and feasibility trials, Billingham et al [43] reported a median sample size of 36 participants, ranging from 10 to 300 participants. The aim of the SafePlan trial is to evaluate the feasibility of using the app as an adjunct to treatment. According to the clinician’s and service manager’s advice, a sample of 60 patients would be eligible for recruitment. With an expected 25% (20/80) dropout rate [43], a sample of 80 patients appeared adequate for the purposes of this study. Therefore, we will aim to recruit 80 patients from mental health services in Health Service Executive (HSE) West and Mid-West.

### Setting

Participants will be recruited from community mental health services across 2 Irish community health care organizations. Participants will complete the baseline, postintervention, and follow-up measures in their mental health service setting. The orientation to the SafePlan intervention will be conducted face-to-face in the participant’s service. The control condition participants will also be introduced to paper-based safety planning face-to-face and in their service. Semistructured interviews will be conducted in the participants’ service and in the clinician’s workplace. Interviews exploring the intervention process may also be conducted by phone, depending on participant’s preferences and other safety considerations. Where face-to-face data collection is not possible owing to health concerns or other unforeseen circumstances, measures may be completed remotely. In such instances, the researcher will communicate with the participant’s key worker and members of the treating team, following the call to ensure that any information relevant to the participant’s safety is communicated to their treating team.

### Procedures

Mental health service multidisciplinary teams across both health care areas will be informed of the study and made aware of the inclusion and exclusion criteria. Team members will be asked to identify patients who are eligible to participate and for whom a safety plan is clinically indicated. Mental health service professionals will inform potential participants about the study and invite them to meet the researcher. If the patient agrees, the researcher will approach the patient, provide them with a participant information sheet, and answer any questions they may have. If the patient agrees, informed consent will be obtained by the researcher. In consenting to participate, participants will also agree to the researcher accessing their medical file notes. Information on the semistructured interviews and fidelity assessment will also be provided, and consent to future contact for this purpose will be sought. Clinicians across both community health organizations will be invited to participate in semistructured interviews to discuss their experiences of delivering the intervention.

Previously established clinician champions at each site will promote recruitment within their own teams and at weekly team meetings. Doctorate in clinical psychology students, in fulfillment of their clinical research experience and under the supervision of the principal investigator, will also support data collection across sites.

Baseline assessment measures will be administered to eligible participants who have consented to participate. Following the baseline assessment, participants will be randomized (1:1) to either the SafePlan or control condition.

**Figure 1:**
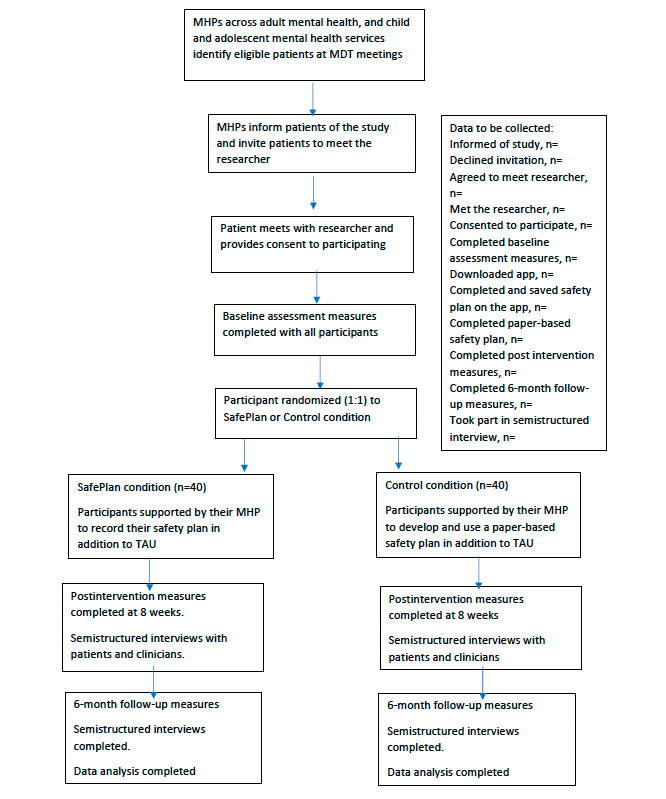
Overview of SafePlan protocol. MDT: multidisciplinary team; MHP: mental health professional; TAU: treatment as usual.

An overview of the study procedures is provided in Figure 1.

### Randomization

Randomization of participants will occur with equal probability for both groups. Participants will be stratified based on the suicide outcomes reported using the C-SSRS (Suicidal ideation/Non Suicidal Self Injurious Behavior/Suicide Attempt). Randomization will be carried out by a clinician using the sealed envelope randomization package.

### Blinding

Neither the participant nor the researcher will know the treatment arm allocation while the initial study measures are being completed. Participants will receive either the SafePlan as an adjunct to TAU or TAU supplemented with a paper-based safety plan. After randomization, both the participant and researcher will be unblinded to the participant’s allocation, which is unavoidable given the nature of the study.

Before starting the SafePlan intervention, all participants will receive a 15-minute training session on how to use the app, including downloading and password protection settings. Measures will be taken face-to-face at baseline, post intervention (8 weeks), and at 6-month follow-up. In addition to accessing TAU, phone-based check-ins will be conducted at week 4 for all participants. Participants will, therefore, have face-to-face or telephone contact with the clinician researcher at baseline assessment, 4 weeks (check-in), 8 weeks (post intervention), and 6-month follow-up. The control group participants will receive the same schedule of contacts and assessment measures.

### SafePlan Condition

The SafePlan mobile app is available to download for free and is accessed at no cost to participants. SafePlan enables participants to record their safety plan, developed with a clinician, and store it on the app to facilitate access and use outside of the clinic. Participants assigned to the SafePlan condition will meet the researcher for instructions on downloading and using the app on their personal smartphones and subsequently meet their key clinician to guide individual use of the app for safety planning. Participants will be guided by the researcher and subsequently their clinician to use SafePlan to record their safety plan in the clinic and implement the plan outside the clinic. Both patients and clinicians will be encouraged to refer to the SafePlan app during their appointments over the 8-week course of the intervention period. The SafePlan app will be used as an adjunct to treatment and will be implemented in parallel with routine community mental health care.

### Control Condition

Participants assigned to the control condition will similarly meet the researcher following enrollment for orientation to paper-based safety planning and subsequently meet their clinician to guide individualized safety planning using the safety planning template by Brown and Stanley [12]. As with the SafePlan condition, participants in the control group will be guided by the researcher and subsequently by their clinician to develop and record a safety plan using the paper-based safety plan template by Brown and Stanley [12]. Both patients and clinicians will be encouraged to refer to the paper-based safety plan during their appointments over the 8-week course of the intervention period. A paper-based safety plan will be used in addition to routine community mental health care.

### Data Collection

#### Feasibility

Feasibility is defined as the extent to which the app can be successfully used by patients attending community mental health services and by their clinician as components of standard treatment. Feasibility will be assessed in terms of recording a safety plan on the app, in-session app use, and app use over the course of treatment (as reported by patients and clinicians). Quantitative data from patients will be collected at baseline, post intervention (8 weeks), and at 6-month follow-up; qualitative data from patients and clinicians will be collected through semistructured interviews. Table 1 presents the study data collection timelines. Psychometric measures will be administered at multiple time points to identify possible differences in the outcomes over the course of the study.

Feasibility outcomes will include the acceptability of the intervention to participants and clinicians, feasibility of delivery in this setting, recruitment, retention, and app use. Quantitative data on the number of potential patients who were approached and who declined, who were not eligible, and who were retained will be recorded and presented in the Consolidated Standards of Reporting Trials diagram for the study.

This study will be conducted in line with the MRC guidelines on the evaluation of complex interventions [37], with data collected via quantitative and qualitative methods.

Participants who consent to be contacted for the qualitative element of the study will be invited to participate in a one-to-one interview about their experiences of the intervention and of study procedures. The interviews will be conducted face-to-face at the university or health service site, and where necessary, participants will be given the additional option of a telephone interview. In the case of telephone interviews, verbal consent will be audio-recorded at the beginning of the interview.

Mental health professionals (n=12) directly involved in participants’ care and in delivering the intervention (eg, CAMHS/AMHS multidisciplinary team members) will be invited to participate in the semistructured interviews. Interviews will focus on the feasibility of the intervention and the acceptability of the study and intervention procedures, including experienced or perceived barriers and facilitators, intervention “fit” within the setting, and suggestions for improvement.

**Table 1:** Study data collection timelines.

	Demographic information	Clinical file data	Client Service Receipt Inventory	Columbia Suicide Severity Rating Scale	Beck Scale for Suicide Ideation	Interpersonal Needs Questionnaire	Coping Self-Efficacy Scale	Safety plan completion	App use (self-report)	Paper plan use (self-report)
Baseline SafePlan condition	✓	✓	✓	✓	✓	✓	✓			
Baseline control condition	✓	✓	✓	✓	✓	✓	✓			
4-week check-in SafePlan condition				✓				✓	✓	
4-week check-in control condition				✓				✓		✓
8-week SafePlan condition			✓	✓	✓	✓	✓	✓	✓	
8-week control condition			✓	✓	✓	✓	✓	✓		✓
6-month SafePlan condition			✓	✓	✓	✓	✓		✓	
6-month control condition			✓	✓	✓	✓	✓			✓

#### Outcome Measure Feasibility

Participants will be asked to complete several questionnaires with the researcher to assess the feasibility and acceptability of using these questionnaires in a full trial. The questionnaires to be completed during the trial are detailed below (an additional check-in at 4 weeks will include further administration of the C-SSRS [38]).

The BSSI [39], the proposed primary outcome for a full trial, will be administered at baseline, post intervention (8 weeks), and at 6-month follow-up to assess the feasibility of this measure in collecting suicide outcome data at multiple time points. The data collected using the BSSI will be used to calculate the sample size required for a full trial. The scale has been used as a tool for screening for the presence or absence of suicidal ideation and, more recently, as a brief measure of change in suicidal thoughts and ideation over time. This scale has high internal reliability and concurrent validity [39].

The C-SSRS is a 20-item semistructured brief and valid tool used to assess suicide risk including suicidal ideation, suicidal behaviors, previous attempts, and preparatory behaviors [38].

The CSES has shown reliability and validity in a mental health service population and can be used to assess changes in coping ability over time [40]. The Cronbach α coefficients of the factors 1, 2, and 3 and the whole CSES-Vietnamese version were at acceptable levels (.91, .86, .75, and .93, respectively).

The INQ is a 15-item measure of perceived burdensomeness and thwarted belongingness, with items rated on a 7-point Likert-type scale [41].

The CSRI is a measure used in mental health service evaluation to capture information about the use of services. The following domains will be captured within this study: general practitioner (GP) consultations, outpatient visits, use of hospital services for physical or mental health problems, mental health helpline contacts, and psychiatrist or social worker or psychologist contacts [42].

The remaining data to be gathered at 8 weeks will include the self-rated helpfulness, benefit, ease of use, likelihood of continued use, and likelihood of recommending the SafePlan app to others.

#### Demographic Data

Participants will be asked to provide consent to share clinical data and will have demographic data collected at enrollment. This will include participant age, sex, relationship status, highest level of educational attainment, employment status, type of service (AMHS or CAMHS), and geographic service area (Community Health Organisation 1 & 2). Clinical data collected from the participant’s file will include details of current psychiatric diagnoses, current treatment plan (including medication if relevant), key clinician profession, and information in relation to previous suicidality to supplement participant self-report (suicidal ideation, nonsuicidal self-injurious behavior, and previous suicide attempt). Information regarding previous suicide risk or behavior is necessary to support patient safety and to inform factors that may be considered potential moderators or mediators in a full trial. A description of treatment received in both the TAU and the SafePlan conditions will be captured using the CSRI and clinical file data.

#### Data Sharing

At baseline, participants will be asked to consent to the researcher accessing their mental health clinical file; the researcher accessing details of the participant’s emergency contact or key clinician, consultant psychiatrist, and GP and sharing clinically important information with those individuals to maintain patient safety as necessary during the trial; and the collection of data post intervention and at 6-month follow-up.

Participants in the SafePlan condition will hold their own data on their device until they choose to show it to their mental health professional when they attend the clinic in person. Their data are not automatically shared or monitored, and this is communicated to participants both upon enrollment in the study and through the terms and conditions presented when downloading the app.

A separate participant information and consent form has been developed for use with mental health professionals who will partake in semistructured interviews focusing on the feasibility and acceptability of the intervention and study procedures. All interview data will be anonymized in the final report.

### Qualitative Interviews

Qualitative interviews will be conducted with patients (SafePlan group, n=12; control group, n=12) and clinicians (n=12) involved in delivering the intervention to assess the feasibility of the intervention and study procedures. The participants and their clinicians will be informed of the qualitative component of the trial during the consent process. Participants will be invited to attend a semistructured interview following the completion of the 8-week intervention. Semistructured interview topic guides will be used. Interviews will seek to better understand participants’ experience of the SafePlan intervention and of the study procedures and to identify barriers to engagement with the intervention. Researchers will also seek to interview participants in the control condition (n=12) to better understand their experiences of TAU, paper-based safety planning, potential contamination, and the acceptability of study procedures.

### Ethics Approval

This study was approved by the University Hospital Limerick Research Ethics Board in October 2021 (REC Ref: 055/2021). This study was preregistered through the Open Science Framework.

### Consent

The researcher will follow the SafePlan Standard Operating Procedure to take appropriate action to maintain participant safety throughout. In accordance with the HSE CAMHS Operational Guidelines [44] and local HSE policy and as stated in the study information sheet, this may include contacting the participant’s key clinician, consultant psychiatrist, GP, and emergency contact or contacting emergency services.

Participants will be identified by members of their mental health team and will be accessing treatment with an assigned key therapist for the entire duration of the intervention. Clinicians will identify potential participants for whom a safety plan is clinically indicated and will provide them with further information about the trial. Participants who wish to participate will be approached by the researcher, and informed consent will be obtained. In the case of those aged <18 years, assent will be required from the young person, and parental or legal guardian signed consent will be obtained before the person can proceed to enrolling in the study. All participants will be informed of their right to refuse to participate and their right to withdraw from the study.

In consenting to participate, the participants will also agree to the researcher accessing their clinical file. Information on the semistructured interviews and the fidelity assessment will also be provided, and consent to future contact for these purposes will be sought.

Data collected during the administration of the outcome measure will be shared with the participant’s key clinician to support participant safety. The researcher and any personnel involved in the study will receive standardized training in addressing suicidality in line with the Irish HSE National Office for Suicide Prevention Education and Training Plan 2021-2022 [45]. Baseline, outcome, and follow-up assessments will be conducted (unless otherwise required by the participant) in the participant’s mental health clinic and face-to-face to ensure optimum clinical support in situ. It is hoped that the semistructured interviews will provide further information on any potential adverse effects of data collection.

The SafePlan study will be conducted in accordance with the International Council for Harmonisation of Technical Requirements for Pharmaceuticals for Human Use Good Clinical Practice standards, which is an international minimum quality standard for the ethical and scientific conduct of clinical research [46]. Safety planning is an established suicide prevention intervention that has been found to reduce the recurrence of suicidal behaviors [15]. Therefore, although the information arising from that intervention will be recorded differently, participants across conditions will receive an evidence-based intervention that is considered good practice alongside TAU. One group will store this information on a paper-based safety plan template, and the other group will store the information on their SafePlan app.

### Fidelity Assessment

The MRC guidelines [37] on complex interventions strongly recommend the inclusion of fidelity assessment to explain discrepancies between expected and observed outcomes, to understand how context influences outcomes, to clarify causal mechanisms, and to provide insights to aid implementation.

Treatment fidelity assessment will include the following:

1. Documentation of key clinician’s behavior, including attendance at the SafePlan orientation and training session, completion and filing of paper-based safety plan (control condition), and development of an interactive safety plan and recording this plan using the SafePlan app (intervention condition).
2. A checklist based on the 6 core steps outlined in the safety planning template by Brown and Stanley [13] will be used to assess the extent to which key components of safety planning are addressed by clinicians in both conditions.
3. A sample (16/80, 20% approximately) of participant intervention sessions will be attended by the researcher, and the checklist will be completed to further assess the fidelity of the plan by Brown and Stanley [13].

### Health Economics

A cost-outcome description will be undertaken. Data on costs will be collected on resource use related to intervention delivery (staff costs, training, and intervention materials) and patient resource use in terms of the broader health service. The CSRI will be used at baseline, 8 weeks, and 6-month follow-up to capture information regarding the use of health and social care services, such as GP consultations, outpatient visits, use of hospital services for physical or mental health problems, and social worker contacts.

The cost of providing the intervention will be calculated based on the average time taken to carry out the intervention and the hourly cost of employing the mental health professional involved. The hourly employment rate of a mental health professional at the midpoint of the salary scale will be calculated based on the available health service salary scale [47]. This will be revised upwards to account for employer-related costs and overheads, based on guidance for conducting an economic analysis within the Irish health care system [48]. Following estimation of the cost of carrying out the intervention, net cost benefit and cost-benefit ratio for providing the service will be calculated.

### Data Analysis

#### Quantitative Data Analysis

Descriptions of recruitment rates, attrition, retention, and reported app use will be summarized. The baseline characteristics of both groups will be summarized (age, diagnosis, and treatment details). Descriptive statistics will be used to examine the uptake of the app (reported in-session app use, reported app use outside the clinic, completion of a safety plan using the app, and use of modified exponential moving average features). Data from all other measures completed at baseline, post intervention, and at 6-month follow-up (C-SSRS, INQ, Emotion Regulation Questionnaire, and Client Receipt Inventory) will be summarized.

The proposed primary outcome measure to be used in a further definitive trial—BSSI—will be summarized by a randomized group. Analyses will be conducted according to the intention-to-treat principle, using available measurements from all participants and according to their allocated groups. Missing data will be accommodated based on all available information using maximum likelihood estimation. We will examine between-group differences in BSSI scores from baseline to post intervention (8 weeks). To further explore preliminary efficacy, we will evaluate within-group changes from baseline to post intervention for each condition separately and then for both groups combined. Finally, within each condition separately, we will explore the potential maintenance of intervention benefits with a repeated measures ANOVA, including the 3 data collection time points (baseline, 8 weeks, and 6 months).

#### Qualitative Data Analysis

Qualitative data from interviews with study participants and clinicians will be analyzed via thematic analysis using the approach by Braun and Clark [49]. Data collection will continue until data saturation is reached. In total, 25% (9/36) of the interviews will be double coded to ensure reliability. Disagreements will be resolved by discussion.

There are specific ethical challenges when conducting qualitative research. As there may be potential for distress, the team developed a research protocol that provides contact details of appropriate additional support if needed. It is important to maintain confidentiality and pseudoanonymity. Informed consent is an ongoing process that continues after obtaining written consent [50]. The team will be cognizant of this throughout the project.

McGrath and Nilsson [51] outlined the challenges in qualitative data sharing. However, transparency and rigor are critical. We will use some of the strategies outlined by McGrath and Nilsson [51], which include outlining procedures for data sharing when obtaining informed consent, data minimization, and pseudonymization.

Tsai et al [52] advocated that “coding queries may offer greater anonymity compared with full transcripts because statements are grouped by theme rather than by study participant.” They further support this process as preferable to sharing full transcripts. In this research, qualitative analysis will be managed using NVivo (version 12; QSR International) qualitative analysis software [53]. This will provide a clear audit trial and permit the execution of coding and matrix queries to ensure transparency.

### Progression From Feasibility to Full Trial

Shanyinde et al [54] reported 14 issues that needed to be evaluated in feasibility studies or pilot trials. “A process for Decision-making after Pilot and feasibility Trials” (ADePT) by Bugge and Williams [55] examines the “methodological issues” identified in a feasibility study that may hinder or facilitate progression to a definitive trial. ADePT permits an examination of the evidence from the feasibility study and the degree to which “methodological issues, ” such as recruitment, retention, adherence, and acceptability of the intervention, among others, have been successful. From the feasibility study findings, ADePT provides a framework to decide whether there should be an amendment to the intervention, context of its delivery, and confirmed trial design before embarking on a full trial. This study will use the ADePT 14-point reporting framework of methodological issues and decision tools to evaluate whether the feasibility study has achieved its objectives and to identify the need for intervention, clinical context, and trial design modification.

## Results

As of January 2023, funding has been approved, ethics approval has been received, and clinician champions across mental health service sites have been established. Service-wide Suicide Prevention and Self-harm Mitigation Training has been completed with clinicians with the support of the National Office for Suicide Prevention. The process of research personnel recruitment has commenced. Data collection is expected to commence when personnel are in post (estimated April 2023). The projected date of submission of completed manuscript is April 2025.

## Discussion

### Principal Findings

This study will provide clinically valid data on the feasibility and acceptability of the SafePlan app as an adjunct to therapy for patients (aged 16-35 years) accessing community mental health services and their clinicians. It is hypothesized that patients and clinicians will find the app both feasible and acceptable and that important barriers and enablers to its use within Irish community mental health services will be identified. Data regarding the feasibility of the study procedures for both patients and clinicians will inform the decision to progress to a full trial. This is the first study to examine the feasibility of the SafePlan app and accompanying trial study procedures. Suicide outcome data collected at baseline, post intervention, and at 6-month follow-up will inform the sample size required for a full RCT. These data will also help determine whether the SafePlan app condition yields superior outcomes on standardized measures of suicidal ideation, suicidal behavior, and nonsuicidal self-injurious behavior at 6-month follow-up and post treatment when compared with the control condition. It is hypothesized that the SafePlan app condition will yield superior outcomes when compared with the control condition.

Safety planning interventions offer an evidence-based means of addressing suicidality in at-risk individuals [14]. A meta-analysis of safety planning–type interventions (SPTIs) for suicide prevention found that the relative risk of suicidal behavior among patients who received an SPTI compared with a control condition was 0.570 (95% CI 0.408-0.795; *P*=.001; number needed to treat: 55). The results support the use of SPTIs to help prevent suicidal behavior and the inclusion of SPTIs in clinical guidelines for suicide prevention [14]. SafePlan moves beyond the recording of safety plans using paper-based templates to record and store the plan, developed with a health care professional, on a mobile app. The results of this study will inform further research and the integration of safety planning apps within mental health services.

SafePlan is distinctly different from the mobile apps currently being trialed with this population [19,31,32]. Previous and ongoing trials used a safety planning app and a separate ecological momentary assessment app in parallel. Trial authors note “the next step would be to combine mobile safety-planning and self-monitoring apps” [19]. SafePlan delivers this by providing a means of recording behaviors that patients can discuss with their mental health professional at their next clinic appointment. The findings of this study will, therefore, provide provisional data on the feasibility and acceptability of this next-generation safety planning app in community mental health services.

Previous research has focused on the use of safety planning interventions in hospital settings and as a stand-alone intervention [13]. However, safety planning is also an important treatment component of community mental health services for those presenting with suicidal ideation and behaviors. This study adds to the existing research by focusing on the feasibility of using such technology within such services.

### Limitations

A potential limitation of the study procedure is that the participants will be unblinded to the type of intervention once they have been assigned to either condition. This is unavoidable, given the distinct nature of each condition (paper based vs app based). However, both the participants and researchers will be unaware of the treatment condition while the baseline measures are being completed.

As data are not automatically shared by the app with the clinician on an ongoing basis, the researcher will depend on the participant attending follow-up appointments and choosing to share their data at that time. It may be the case that useful data will be lost because of attrition, particularly at 6-month follow-up. This may be particularly relevant to understanding the experiences of participants who discontinued using the app. However, the data-sharing procedures were designed to respect and protect participant data. All participants were informed of the procedures before they consented to participate. It is hoped that the inclusion of semistructured interviews with both patients and their clinicians will help address this potential limitation.

### Conclusions

Health care policy nationally and internationally [56,57] proposes the need for integrated care facilitated by innovations in digital health. The use of mobile devices by individuals at risk of suicide has been recommended by the World Health Organization [16]. The SafePlan app was designed to support individuals in completing and recording an evidence-based safety plan with their clinician. This protocol outlines the first feasibility and acceptability trial of the SafePlan App and the related study procedures. The trial adds to the existing research conducted in hospital settings by examining the feasibility of this safety planning mobile app within community mental health services. The findings may have implications for patients, clinicians, health services, and policy makers regarding the integration of safety planning mobile apps more generally and may inform future research in mHealth and suicide prevention.

## Abbreviations

ADePT: Decision-making after Pilot and feasibility Trials
AMHS: adult mental health services
BSSI: Beck Scale for Suicide Ideation
CAMHS: child and adolescent mental health services
CSES: Coping Self-Efficacy Scale
CSRI: Client Service Receipt Inventory
C-SSRS: Columbia Suicide Severity Rating Scale
DBT: dialectical behavior therapy
GP: general practitioner
HSE: Health Service Executive
INQ: Interpersonal Needs Questionnaire
mHealth: mobile health
MRC: Medical Research Council
RCT: randomized controlled trial
SPI: safety planning intervention
SPTI: safety planning–type intervention
TAU: treatment as usual

## Data Availability

Deidentified aggregate data sets generated during or analyzed during this study may be made available from the corresponding author upon reasonable request and provided data sharing is in accordance with the study’s ethics approval, the study’s data protection impact statement, and general data protection regulations.
